# Genome Sequencing of *Rahnella victoriana* JZ-GX1 Provides New Insights Into Molecular and Genetic Mechanisms of Plant Growth Promotion

**DOI:** 10.3389/fmicb.2022.828990

**Published:** 2022-03-30

**Authors:** Wei-Liang Kong, Wei-Yu Wang, Sheng-Han Zuo, Xiao-Qin Wu

**Affiliations:** ^1^Co-Innovation Center for Sustainable Forestry in Southern China, College of Forestry, Nanjing Forestry University, Nanjing, China; ^2^Jiangsu Key Laboratory for Prevention and Management of Invasive Species, Nanjing Forestry University, Nanjing, China

**Keywords:** complete genome sequence, *Rahnella victoriana*, biofertilizer, volatile organic compounds, natural field environment

## Abstract

Genomic information for bacteria within the genus *Rahnella* remains limited. *Rahnella* sp. JZ-GX1 was previously isolated from the *Pinus massoniana* rhizosphere in China and shows potential as a plant growth-promoting (PGP) bacterium. In the present work, we combined the GridION Nanopore ONT and Illumina sequencing platforms to obtain the complete genome sequence of strain JZ-GX1, and the application effects of the strain in natural field environment was assessed. The whole genome of *Rahnella* sp. JZ-GX1 comprised a single circular chromosome (5,472,828 bp, G + C content of 53.53%) with 4,483 protein-coding sequences, 22 rRNAs, and 77 tRNAs. Based on whole genome phylogenetic and average nucleotide identity (ANI) analysis, the JZ-GX1 strain was reidentified as *R. victoriana*. Genes related to indole-3-acetic acid (IAA), phosphorus solubilization, nitrogen fixation, siderophores, acetoin, 1-aminocyclopropane-1-carboxylate (ACC) deaminase, gamma-aminobutyric acid (GABA) production, spermidine and volatile organic compounds (VOCs) biosynthesis were present in the genome of strain JZ-GX1. In addition, these functions were also confirmed by *in vitro* experiments. Importantly, compared to uninoculated control plants, *Pyrus serotina*, *Malus spectabilis*, *Populus euramericana* (Dode) Guinier cv. “San Martino” (I-72 poplar) and *Pinus elliottii* plants inoculated with strain JZ-GX1 showed increased heights and ground diameters. These findings improve our understanding of *R. victoriana* JZ-GX1 as a potential biofertilizer in agriculture.

## Introduction

According to the latest statistics of the United Nations, the global population is expected to reach 9.7 billion by 2050 (Goswami and Deka, 2020). To meet the growing demand for food, excessive use of chemical fertilizers and pesticides in agricultural production has damaged soil health and ecology (Ruzzi and Aroca, 2015). At the same time, abiotic stresses, including drought, high salinity, and toxic heavy metals are prevalent worldwide (Rosier et al., 2018; Mondal et al., 2021). It is estimated that abiotic stresses affect approximately 10 hectares of land per minute, with three hectares affected by soil salinization around the world (Ha-Tran et al., 2021). Similar to high salinity, extremely dry conditions lead to an annual harvest loss of 17% of harvests in tropical, arid and semiarid areas (Chandra et al., 2021). Trees suffer even more from the recurrence of abiotic stress owing to their long lifecycle and the global forest cover is gradually decreasing (Teshome et al., 2020). Therefore, appropriate measures need to be taken to make the whole agricultural and forestry ecosystem develop in the direction of sustainable intensification (Pretty et al., 2018).

With progress in the development of genetic tools and technology, our understanding of the “black box” of microorganisms in soil has become more transparent, and we are at a moment when rhizosphere microbial research is likely to be of great application value (Olenska et al., 2020). A large number of studies have determined the important contributions of specific rhizosphere growth-promoting bacteria to sustainable agricultural production. The plant growth-promoting rhizobacteria (PGPR) reported to date are mainly concentrated in *Bacillus*, *Pseudomonas*, *Enterobacter*, *Burkholderia*, *Klebsiella*, *Azospirillum*, and *Serratia* (Liu et al., 2020a; Alexandre et al., 2021; Kusale et al., 2021; Wang et al., 2021; Yasmin et al., 2021). These rhizosphere colonizing microorganisms employ a variety of mechanisms to alleviate adverse soil conditions, thus promoting plant growth. They mobilize soil nutrients by secreting organic acids, siderophores, fixed nitrogen, and dissolved phosphate (Kong et al., 2020a; Liu et al., 2020b; Mariotti et al., 2021). They can also secrete a series of substances, such as 1-aminocyclopropane-1-carboxylate (ACC deaminase), volatiles, spermidine, gamma-aminobutyric acid (GABA), and phytohormones to induce plant resistance to abiotic stress (Zhou et al., 2016b; Liu et al., 2020c; Nascimento et al., 2020; Choudhury et al., 2021). Up to now, PGPR strains have been studied in detail in agricultural crops, but their application in woody plants has not been well-explored.

Strain JZ-GX1 was screened with a high yield phytase as the index in our laboratory and was first classified as *Rahnella aquatilis* based on the 16S rRNA gene sequence. However, with the advancement of taxonomic research, many members of *Rahnella* have been gradually reported, so it is necessary to identify strain JZ-GX1 at a greater resolution. Previous studies have reported that the JZ-GX1 strain exhibited a significant growth-promoting effect on poplar and corn in greenhouse conditions (Li et al., 2013, 2021), so does it have the characteristics of plant growth-promoting in addition to the degradation of phytate? Moreover, the growth-promoting effect of this strain on woody plants in the field needs to be further studied. Therefore, the whole genome sequencing technique was used in this study to provide new insights into the molecular and genetic mechanism of *Rahnella* sp. JZ-GX1 in promoting plant growth. Furthermore, two ecologically important trees and two economic trees were selected for use in field experiments to further explore the growth-promoting ability of strain JZ-GX1 under natural conditions. The overarching goal was to assess this strain as a new microbial inoculant for sustainable agricultural practices.

## Materials and Methods

### Bacterial Strain and DNA Preparation

*Rahnella* sp. JZ-GX1 was isolated from the rhizosphere soil of a 28-year-old *Pinus massoniana* in Guangxi, China, on April 8, 2011, and was deposited in the Chinese Center for Type Culture Collection (Accession No. CCTCC M2012439). This strain was routinely incubated in Luria-Bertani (LB) liquid media at 28°C with shaking for 24 h. Genomic DNA was extracted using a modified freeze–thawing method (Chen et al., 2020).

### Genome Sequencing, Assembly, and Annotation

*Rahnella* sp. JZ-GX1 genome was sequenced by the single molecule real-time (SMRT) method at Biomarker Technology Co., Ltd. (Beijing, China). The genome assembly was created using Nanopore long reads, and Illumina paired-end sequences were used for base and indel correction. The filtered subreads was assembled using Canu v1.5 software. Finally, the Pilon software is used to further correct the assembled genome using second generation data, and the final genome with high accuracy was obtained.

The predicted gene sequences were compared with the Clusters of Orthologous Groups (COG), Kyoto Encyclopedia of Genes and Genomes (KEGG), Swiss-Prot, TrEMBL, non-redundant (Nr), and other functional databases by BLAST, and gene functional annotations were obtained (Tatusov et al., 2003). Based on the results from comparison with the Nr database (Aziz et al., 2008), the application software Blast2GO was used to annotate functions according to the GO database. HMMER software was applied for functional annotation based on the Pfam database (El-Gebali et al., 2019). In addition, the functions of genes were annotated and analyzed using COG and KEGG metabolic pathway enrichment analysis.

### Phylogenetic Tree Construction and Average Nucleotide Identity Analysis

A phylogenetic tree was constructed by combining the genome of JZ-GX1 with a set of closely related genomes selected from all public KBase genomes using the Insert Genome Into Species Tree 2.1.10 tool.^1^ Relatedness was determined by alignment similarity with a selected subset of COG domains. Next, a phylogenetic tree was reconstructed using FastTree (version 2.1.10). Average Nucleotide Identity (ANI) analysis was performed between strain JZ-GX1 and other *Rahnella* isolates included in the phylogenetic tree using an online ANI calculator.^2^

### Nucleotide Sequence Accession Numbers

The chromosome, plasmid 1 and plasmid 2 sequences are available under NCBI BioProject PRJNA720502, with accession numbers CP089919, CP089920, and CP089921, respectively.

### Assessment of Indole-3-Acetic Acid and 1-Aminocyclopropane-1-Carboxylate Deaminase Activity

To induce the production of indole-3-acetic acid (IAA), 250 μL of a culture of strain JZ-GX1 grown overnight in LB medium was transferred into 25 mL of TSB medium supplemented with sterile-filtered L-tryptophan (500 μg/mL). The liquid culture was grown at 28^°^C and 180 rpm, and cells were separated from the exhausted medium by centrifugation (10,000 × *g* for 15 min). The concentration of IAA in the bacterial solution was sampled and determined at 96 h after inoculation. The collected supernatant was filtered through 0.22 μm cellulose acetate filters (DISMIC^®^; Frisenette ApS, Knebel, Denmark). The bacterial supernatant (1 mL) was mixed with 4 mL of Salkowski’s reagent (50 mL of 35% HClO_4_, 1 mL of 0.5 M FeCl_3_) and allowed to rest for 30 min in the dark. After incubation, absorbance was measured at 530 nm (pink color) (T60 UV-VIS Spectrophotometer; PG Instruments, Leicester, United Kingdom) and quantified using the calibration curve of an IAA standard with linear regression analysis (Kang et al., 2020). The ACC deaminase activity was determined by previously described method (Penrose and Glick, 2003). The experiment was repeated two times, and each treatment was conducted in triplicate.

### Evaluation of Siderophore Production, Nitrogen Fixation, Phosphorus, and Potassium Solubilizing Ability

For siderophores production, strain was cultivated in a Chrome Azurol-S agar assay (Schwyn and Neilands, 1987), and positive results were indicated by the formation of a clear orange zone around the colonies. *Burkholderia pyrrocinia* JK-SH007 was used as a positive control.

Nitrogen fixation ability using N-free Ashby medium agar plates contained 5 g of glucose, 5 g of mannitol, 0.1 g of CaCl_2_⋅2H_2_O, 0.1 g of MgSO_4_⋅7H_2_O, 0.005 g of Na_2_MoO_4_⋅2H_2_O, 0.9 g of K_2_HPO_4_, 0.1 g of KH_2_PO_4_, 0.01 g of FeSO_4_⋅7H_2_O, 5.0 g of CaCO_3_, 15.0 g of agar in 1 L of distilled water, the final pH was adjusted to 7.3 (Liu et al., 2017).

The rhizosphere bacteria were assessed for potential inorganic phosphate solubilization on the National Botanical Research Institute’s phosphate (NBRIP) growth medium [per liter: 10.0 g of glucose, 5.0 g of MgCl_2_⋅6H_2_O, 0.25 g of MgSO4⋅7H_2_O, 0.2 g of KCl and 0.1 g of (NH_4_)_2_SO_4_] supplemented with Ca_3_(PO_4_)_2_ at a final concentration of 0.5% (Chandran et al., 2014).

Organophosphorus detection medium contained 0.1 g of Calcium phytate, 10.0 g of Glucose, 5.0 g of MgCl_2_, 0.2g of KCl, 0.25 g of MgSO_4_, 0.1 g of (NH_4_)_2_SO_4_, 16.0 g of agar in 1 L of distilled water, the final pH was adjusted to 7.3 (Shen et al., 2016).

Potassium dissolving ability using modified Aleksandrov medium including 5.0 g of glucose, 0.5 g of MgSO_4_⋅7H_2_O, 0.1 g of CaCO_3_, 0.006 g of FeCl_3_; 2.0 g of Ca_3_(PO_4_)_2_, 3.0 g of potassium aluminium silicate and 20.0 g agar in 1 L of distilled water, the final pH was adjusted to 7.2 (Etesami et al., 2017). The JZ-GX1 colonies were stabbed in triplicate on all agar plates using sterile toothpicks and positive results were expressed by the presence of solubilization zone around bacterial colony after 120 h of incubation at 28°C.

Each plate detection was composed of nine replicates, and the experiment was repeated twice.

### Voges-Proskauer Test for Detection of Acetoin

The strain was inoculated in Methyl Red-Voges-Proskauer (MR-VP) broth (glucose 0.5 g, K_2_HPO_4_ 0.2 g, water 1,000 mL, pH 7.2-7.4). After shaker culture at 30^°^C for 2 days, the culture medium was mixed with 40% NaOH, a small amount of creatine was added and the mixture was heated in a boiling water bath to test for a positive reaction (shown as a red color) (Rath et al., 2018). *Escherichia coli* DH5α was used as a negative control. Each treatment was composed of three replicates, and the experiment was repeated twice.

### Detection of Key Genes Related to Plant Growth-Promoting Traits in JZ-GX1

Genomic DNA was extracted using a modified freeze–thawing method (Chen et al., 2020). Then, polymerase chain reaction (PCR) assays were used to detect the *acdS*, *gadB*, *gadD*, *gabT*, *speA*, *speD*, *G1pase*, *pstA*, *pstC*, and *nirB* genes by designing PCR primers based on the genome sequence of JZ-GX1 strain. The primers used in the experiment are listed in Supplementary Table 1.

### Plant-Bacterial Dual Growth Experiments

*Arabidopsis thaliana* Col-0 was used in this study. Seeds were surface sterilized and sown on 1/2 MS agar medium. The Petri dishes were positioned vertically in a growth chamber under a long photoperiod (16 h light/8 h dark) and 70% relative humidity at 22°C. Seven days later, five seedlings were transferred to a new 1/2 MS agar medium, and at the same time, 10 μL of bacterial solution was added to the other compartment, and LB medium without bacterial solution was used as the control. After the bacterial solution was homogeneously distributed in the culture medium, the Petri dish was sealed with parafilm and grew vertically in the same conditions. After 14 days, *A. thaliana* seedlings were removed and weighed with a 1/10,000 balance; the length of the main root of *A. thaliana* was measured and recorded with a Vernier calliper. Then, the seedlings were placed in a Petri dish filled with clear water so that the roots could be fully elongated, and the number of lateral roots was counted and recorded. The root hair of *A. thaliana* was photographed with a Zeiss stereomicroscope (Zeiss Microscope System Standard 16; Carl Zeiss Ltd.; Wetzlar, Germany) (Bhattacharyya et al., 2015; Perez-Flores et al., 2017; Li F. et al., 2020). Each treatment was composed of ten replicates, and the experiment was repeated twice.

### Antagonistic Assay of the Volatile Organic Compounds Produced by *Rahnella* sp. JZ-GX1 Against Plant Pathogenic Fungi

The antifungal effect of the volatile organic compounds (VOCs) produced by *Rahnella* sp. JZ-GX1 was evaluated using I plates (90 mm in diameter). I plates are plastic Petri dishes with a partition in the center that divides the plate into two parts. The microorganisms grown on side thus cannot spread to the other side. However, gas exchange can proceed normally. One compartment of the I plates, containing LB agar medium, was inoculated with *Rahnella* sp. JZ-GX1. The other compartment of the I plates, containing PDA medium, was inoculated with pathogenic fungi using mycelial discs (6 mm diameter). I plates with pathogenic fungi on one half served as the control. The fungal plant pathogens used in this study were preserved in the Forest Pathology Laboratory of Nanjing Forestry University. All plates were sealed with Parafilm and cultured for 5–7 days at 25°C (Zhang et al., 2019c,Zhang et al., 2021). Each treatment was composed of three replicates, and the experiment was repeated twice.

### Field Experiment

The experiment was conducted at nurseries in Changzhou (31°47′N, 119°58′E) and Suqian (33°72′N, 118°68′E), Jiangsu Province. The areas have a subtropical monsoon climate, with an average annual precipitation of approximately 1,056 mm and an annual average temperature of approximately 15°C. The soil type is paddy soil. Two-year-old *Pyrus serotina*, *Malus spectabilis*, and I-72 poplar and 1-year-old *Pinus elliottii* were selected as indicator tree species. Three replicates were applied following in a randomized block design. Each plant was irrigated with 800 mL of bacterial liquid (including 50 mL of bacterial suspension), and seedlings that were not inoculated with JZ-GX1 were used as controls, with 90 plants per treatment. The whole experiment was carried out in open air, with drip irrigation provided when the weather was dry. After 8 months, plant height and ground diameter were recorded.

### Statistical Analysis

Analysis of variance (ANOVA) followed by Duncan’s multiple comparison test was performed for comparisons among all means, and Student’s *t*-test was performed for comparison of pairs of means with SPSS 22.0 software (IBM Inc., Armonk, NY, United States). Error bars show the standard deviation and significance was defined as *p* < 0.01. Graphs were generated using GraphPad Prism 8.0 (GraphPad Software, Inc., United States).

## Results

### General Genome Features and Phylogenetic Analysis of Strain JZ-GX1

**FIGURE 1:**
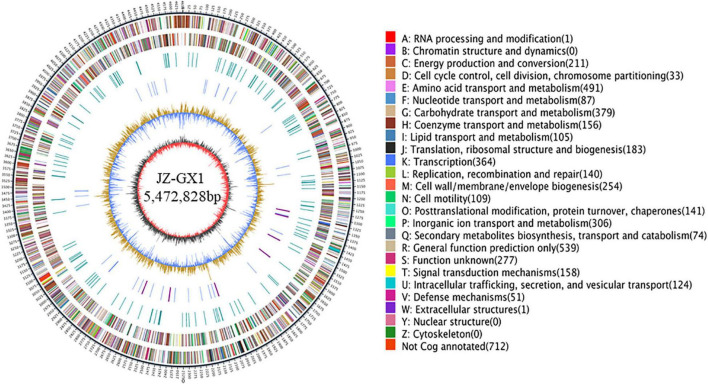
The whole genome of *Rahnella victoriana* JZ-GX1. The genome map is composed of seven circles. From the outer circle to inner circle, each circle displays information regarding the genome of (1) forward CDS, (2) reverse CDS, (3) forward COG function classification, (4) reverse COG function classification, (5) nomenclature and locations of predictive secondary metabolite clusters, (6) G + C content, and (7) GC skew.

The complete genome of *Rahnella* sp. JZ-GX1 contained one gapless circular chromosome of 5,472,828 bp and two plasmids, with a G + C content of 53.53%. Twenty two rRNAs, and 77 tRNAs were found in the chromosome, including 0 pseudogenes, 12 CRISPR sequences, 12 gene islands, and two pre-bacteriophages (Figure 1). A total of 4,483 protein-coding genes were predicted, of which 88.85% were annotated to COG functional categories, 80.68% to GO functional categories and 68.77% to KEGG pathways. Details are presented in Supplementary Table 2.

**FIGURE 2:**
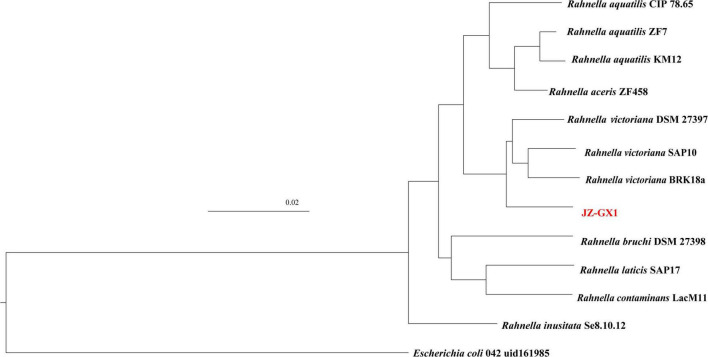
Taxonomic assignment of strain JZ-GX1. *Escherichia coli* 042 uid161985 was used as outgroup. Bar, 0.02 substitutions per nucleotide position.

Phylogenetic analysis based on the whole genome further confirmed the close taxonomic relationship of strain JZ-GX1 with *Rahnella victoriana* (Figure 2). On the basis of ANI, the similarity between genomes of strain JZ-GX1 and other *R. victoriana* strains was approximately 98%; when the strain was compared with other species, the ANI threshold was less than 90% (Table 1). All the data indicated that strain JZ-GX1 belongs to *R. victoriana* rather than *R. aquatilis*, as formerly proposed.

**TABLE 1:** Average nucleotide identity (ANI) values based on alignment of the whole genome of strain JZ-GX1 and the most closely related members of the genus *Rahnella*.

Subject strain	Genome size (bp)	Biosample accession	ANI threshold (%)
JZ-GX1	5,472,828	SAMN18652319	/
*R. victoriana* SAP-10	5,489,796	SAMN16733768	98.87
*R. victoriana* DSM 27397	5,309,719	SAMN10987324	98.92
*R. victoriana* BRK18a	5,563,295	SAMN05249967	98.78
*R. aquatilis* CIP 78.65	5,448,900	SAMN12024752	89.10
*R. aquatilis* KM12	4,878,627	SAMN10579152	89.03
*R. aquatilis* ZF7	5,536,721	SAMN10032142	88.90
*R. aceri*s ZF458	5,602,983	SAMN17207067	88.93
*R. bruchi* DSM 27398	5,501,702	SAMN10095204	87.07
*R. contaminans* Lac-M11	5,230,797	SAMN13909976	85.58
*R. inusitata* Se8.10.12	5,471,205	SAMN16814261	84.45
*R. laticis* SAP-17	5,727,497	SAMN16734072	85.28

### Gene Function Annotation of *Rahnella victoriana* JZ-GX1

**FIGURE 3:**
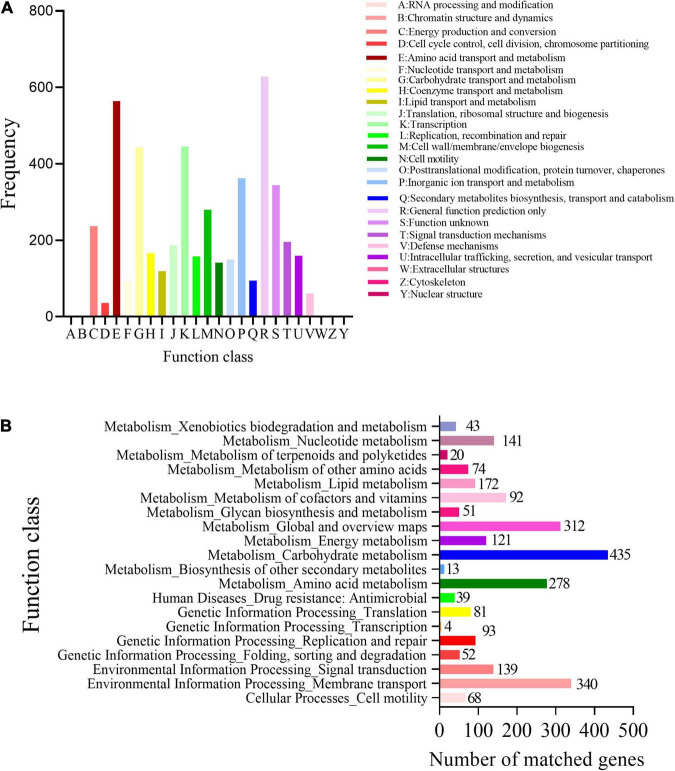
Functional categories of *R. victoriana* JZ-GX1. **(A)** Clusters of Orthologous Groups of proteins (COGs) annotation and **(B)** Clusters of kyoto encyclopedia of genes and genomes (KEGG) annotation.

Based on the COG analysis, the identified proteins were classified into 25 functional categories. Among these proteins, in addition to the high content of COG R (general function), the content of COG E (amino acid transport and metabolism) was the highest, followed by COG K (transcription) and COG G (heredity and basic metabolism), indicating that the functional proteins encoded by the cell genome play an important role in maintaining cellular life and genetic metabolism (Figure 3A).

Using gene KEGG annotations, 20 functional classes were predicted in the genome of *R. victoriana* JZ-GX1, which can be divided into five categories: (1) systems related to cell processes, such as cell movement (68); (2) systems related to environmental information processing, such as signal transduction (139) and transmembrane transport (340); (3) systems related to genetic information processing; (4) systems related to immunity (39); and (5) systems related to metabolism. Among them, metabolism-related systems accounted for the highest proportion of *R. victoriana* JZ-GX1 metabolic pathways (Figure 3B). These results showed that JZ-GX1 strain has strong genetic potential to synthesize secondary metabolites, which is consistent with the above protein annotation results.

### Identification of Genes Responsible for Plant Growth-Promoting Property of *Rahnella victoriana* JZ-GX1

Functional analysis of *R. victoriana* JZ-GX1 genome revealed the presence of several genes contributing directly to plant hormones and nutrient availability. We identified 11 genes encoding key enzymes involved in the synthesis and secretion of IAA through the IPyA (*ipdC*) and IAM (*amiE*) pathways. For the N cycle, genes encoding nodulation protein (*nodN*), nitrate reductase catalytic subunit (*nasA*), and nitrite transporter (*nirC*) were detected. These enzymes are involved in the denitrification process, catalyzing the conversion of nitrate to nitrite to nitric oxide, followed by the conversion of nitric oxide to nitrous oxide. Strain JZ-GX1 possessed genes encoding the synthesis of enterobactin and rhizobactin, which form the main part of catechol and hydroxamic acid type siderophores, and a ferric enterobactin transport system (*fepACDG*) was detected. The annotation information also revealed the presence of several gene clusters involved in mineral phosphate solubilization, including the phosphate-specific transport operon (*pstAC*) as well as a two-component signal transduction system for phosphate uptake consisting of *phoPRH* genes (Table 2).

**TABLE 2:** Genes related to plant growth-promoting activities in the *Rahnella victoriana* JZ-GX1 genome.

Gene ID	Gene name	Gene function
**Auxin biosynthesis**		
GE03900	*trpE*	Anthranilate synthase component I
GE03901	*trpD*	Anthranilate phosphoribosyltransferase
GE03903	*trpB*	Tryptophan synthase subunit beta
GE03904	*trpA*	Tryptophan synthase alpha chain
GE01768	*trpS*	Tryptophanyl-tRNA synthetase
GE00882	*trpR*	Trp operon repressor
GE04238	*mtr*	Tryptophan permease
GE02592	*ipdC*	Indole-3-pyruvate decarboxylase
GE01577	*amiE*	Aliphatic amidase
GE02518	*aec*	Auxin efflux carrier family protein
GE03903	*TSB*	Phosphoribosylanthranilate isomerase
**Nitrogen metabolism**		
GE00696	*nodN*	Nodulation protein
GE03474		Nitrogen fixation
GE01773	*nirB*	Nitrite reductase (NADH) large subunit
GE01772	*nirD*	Nitrite reductase (NADH) small subunit
GE03448	*nasA*	Nitrate reductase catalytic subunit
GE03466	*nrtP*	MFS transporter, NNP family, nitrate/nitrite transporter
GE04729	*nirC*	Nitrite transporter
**Siderophores**		
GE02035	*fes*	Enterobactin esterase
GE00216	*entB*	Isochorismatase
GE02737	*entC*	Isochorismate synthase
GE02242	*fepA*	TonB-dependent receptor
GE02036	*fepC*	Ferric enterobactin transport ATP-binding protein
GE02038	*fepD*	Ferric enterobactin transport system permease protein
GE02037	*fepG*	Ferric enterobactin transport system permease protein
GE03776	*RhbF*	Rhizobactin siderophore biosynthesis protein
GE03029	*CirA*	Catecholate siderophore receptor
GE00738	*FhuD*	Fe^3+^-hydroxamate-binding protein
GE03426	*FhuF*	Ferric iron reductase protein
**Phosphorus metabolism**		
GE00522	*PhoB*	Phosphate regulon transcriptional regulatory protein
GE03390	*PhoH*	Phosphate starvation-inducible protein
GE00521	*PhoR*	Phosphate regulon sensor protein
GE01248	*PstA*	Phosphate transport system permease protein
GE01484	*PstC*	Phosphate transport system permease protein
GE03803	*pqqA*	Alkaline phosphatase
GE04682	*pqqB*	Pyrroloquinoline quinone biosynthesis protein
GE04681	*pqqC*	Pyrroloquinoline quinone biosynthesis protein
GE04680	*pqqD*	Pyrroloquinoline quinone biosynthesis protein
GE04679	*pqqE*	Pyrroloquinoline quinone biosynthesis protein
GE04678	*pqqF*	Pyrroloquinoline quinone biosynthesis protein
GE01596		4-Phytase activity
GE00546	*G1Pase*	Glucose-1-phosphatase

### Gene Mining for Improving Plant Stress Resistance by *Rahnella victoriana* JZ-GX1

In addition to the genes that directly promote growth, many genes responsible for synthesis and transport of compatible solutes were predicted, such as Na^+^/H^+^ antiporter (*NhaAB*), glycine betaine transporter (*opuD*), proline/betaine transporter (*proP*), and exopolysaccharide production protein. Genes encoding acetolactate synthase (*alsD* and *alsS*) and 2,3-butanediol (*bdh* and *budABC*), which are involved in the synthesis of acetoin, a volatile molecule associated with plant growth promotion, were detected in the genome. The gene *gadB* responsible for GABA production, as well as *gabT* and *gabD*, encoding GABA aminotransferase and succinate-semialdehyde dehydrogenase involved in GABA degradation, were predicted. Moreover, arginine decarboxylase (*speA*), agmatinase (*speB*), S-adenosylmethionine decarboxylase proenzyme (*speD*) and spermidine synthase (*speE*), related to spermidine biosynthesis, were present in *R. victoriana* JZ-GX1 (Table 3).

**TABLE 3:** Genes related to plant stress resistance improving in the *Rahnella victoriana* JZ-GX1 genome.

Gene ID	Gene name	Gene function
**Salt tolerance**		
GE00864	*NhaA*	Na^+^/H^+^ antiporter
GE03750	*NhaB*	Na^+^/H^+^ antiporter
GE02187	*opuD*	Glycine betaine transporter
GE02874	*dhaS*	Betaine-aldehyde dehydrogenase
GE02188	*opuBA*	Glycine/betaine ABC transporter ATP-binding protein
GE04729	*proP*	Proline/betaine transporter
GE03756	*eps*	Exopolysaccharide production protein
**ACC deaminase**		
GE03645	*acds*	1-aminocyclopropane-1-carboxylate deaminase activity
**GABA production**		
GE04695	*gadB*	glutamate decarboxylase
GE04546	*gabD*	Succinate-semialdehyde dehydrogenase
GE04524	*gabT*	Gamma-aminobutyrate: alpha-ketoglutarate aminotransferase
**Spermidine biosynthesis**		
GE00638	*SpeA*	arginine decarboxylase
GE00638	*SpeB*	Biosynthetic arginine decarboxylase
GE00775	*SpeD*	S-adenosylmethionine decarboxylase proenzyme
GE00774	*SpeE*	Spermidine synthase
**Volatile organic compounds**		
GE00932	*alsD*	Alpha-acetolactate decarboxylase
GE00822	*alsS*	Acetolactate synthase
GE04344	*bdh*	(R, R)-butanediol dehydrogenase activity
GE00932	*budA*	Alpha-acetolactate decarboxylase
GE00933	*budB*	Acetolactate synthase, catabolic
GE04344	*budC*	Diacetyl reductase [(S)-acetoin forming]

### Plant Growth-Promoting Rhizobacteria Traits Found in *Rahnella victoriana* JZ-GX1 Genome Are Expressed *in vitro*

**FIGURE 4:**
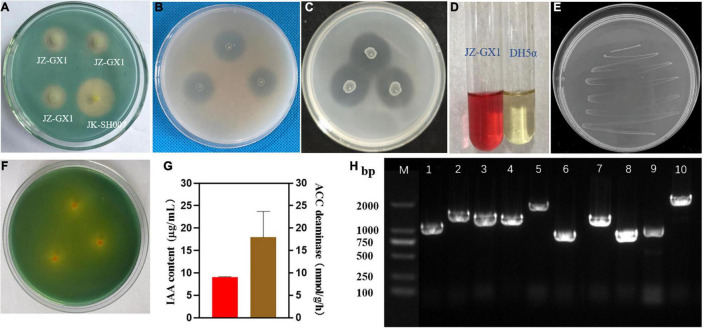
Detection of plant growth-promoting characteristics of *Rahnella victoriana* JZ-GX1. **(A)** Siderophore production, **(B)** inorganic phosphorus dissolution, **(C)** organophosphorus dissolution, **(D)** acetoin production, **(E)** nitrogen fixation, **(F)** potassium solubilization, **(G)** IAA and ACC deaminase production, and **(H)** detection of key gene related to PGP traits in JZ-GX1 by PCR amplification (lane M, DNA marker; 1, *acdS*; 2, *gadB*; 3, *gadD*; 4, *gabT*; 5, *speA*; 6, *speD*; 7, *G1pase*; 8, *pstA*; 9, *pstC*; 10, *nirB*). One-way ANOVA was performed, and Duncan’s *post-hoc* test was applied. Different letters indicate statistically significant differences (*p* < 0.01) among treatments (*n* = 3).

To verify the accuracy of genome sequencing, we investigated the PGP characteristics of strain JZ-GX1 *in vitro*. The results demonstrated that *R. victoriana* JZ-GX1 appeared as a yellow halo on the CAS plate and Aleksandrov plate, indicating that it can secrete siderophores and dissolve potassium (Figures 4A,F). The dissolution circle method showed that JZ-GX1 has the ability to degrade insoluble organic phosphorus and inorganic phosphorus at the same time (Figures 4B,C). Moreover, it could grow on Ashby medium, which proved that it has a certain autogenous nitrogen fixation ability (Figure 4E). The VP test showed that *R. victoriana* JZ-GX1 could produce acetoin (Figure 4D). In addition, the IAA content and ACC deaminase activity were 9.0934 μg/mL and 17.9794 mmol α-keto/mg protein/h, respectively (Figure 4G).

We tested the JZ-GX1 strain for the presence of operons for the biosynthesis of ACC deaminase, GABA, and spermidine, the fixation of nitrogen and the dissolution of phosphorus by PCR using specific primers. A fragment of the predicted size for each of these compounds was observed in the DNA of the genome sequence, which indicates that the strain produces these compounds. Fragments of the predicted sizes for *acdS* (∼1,002 bp), *gadB* (∼1,473 bp), *gadD* (∼1,392 bp), *gabT* (∼1,269 bp), *speA* (∼1,983 bp), *speD* (∼795 bp), *G1pase* (∼1,302 bp), *pstA* (∼846 bp), *pstC* (∼957 bp), and *nirB* (∼2,550 bp) were amplified from strain JZ-GX1 DNA (Figure 4H).

### Volatile Organic Compounds Produced by *Rahnella victoriana* JZ-GX1 Can Promote Plant Growth and Inhibit Phytopathogenic Fungi

**FIGURE 5:**
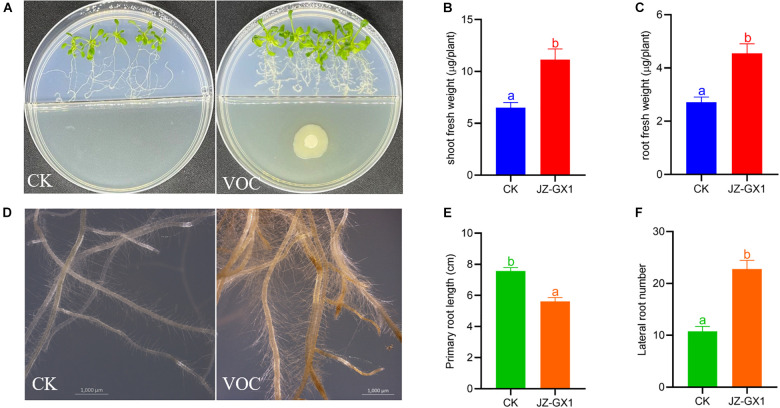
Effect of *Rahnella victoriana* JZ-GX1 VOCs on plant growth and root morphology of *Arabidopsis thaliana* compared to the control (CK). **(A)** Plant phenotype, **(B)** shoot fresh weight, **(C)** root fresh weight, **(D)** root morphology, **(E)** primary root length, **(F)** lateral root length. The scale is 1,000 μm. Different letters indicate statistically significant differences among treatments (Student’s *t*-test, *p* < 0.01) (*n* = 100).

To investigate the effect of VOCs produced by *R. victoriana* JZ-GX1 on plant growth, we performed I-plate assays as these avoid any physical contact between *A. thaliana* and *R. victoriana* JZ-GX1. Biomass production was stimulated in *Arabidopsis* plants exposed to *R. victoriana* JZ-GX1 in this manner (Figure 5A). Following exposure to *R. victoriana* JZ-GX1, the fresh weights of shoots and roots increased significantly by 1.71- and 1.68-fold, respectively, compared to the controls (Figures 5B,C). In addition, *Arabidopsis* exposed to strain JZ-GX1 VOCs showed denser root hairs (Figure 5D). The bacterial VOCs significantly inhibited primary root growth and stimulated lateral root formation at the same time (Figures 5E,F). These data indicate that VOCs from JZ-GX1 can promote plant growth and modulate rhizogenesis in *A. thaliana*.

**FIGURE 6:**
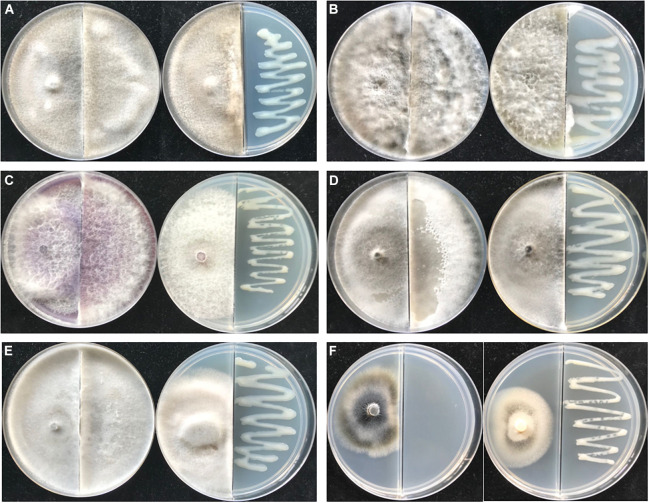
The antifungal spectrum of *Rahnella victoriana* JZ-GX1 VOCs. Plate on the left untreated, plate on the right treated with VOCs. **(A)** *Guignardia camelliae*, **(B)** *Fusicoccus aesculin*, **(C)** *Fusarium oxysporum*, **(D)** *Alternaria tenuissima*, **(E)** *Sphaeropsis sapinea*, and **(F)** *Verticillium dahlia*.

The VOCs emitted by PGPR often show an inhibitory effect on pathogens. As shown in Figure 6, strain JZ-GX1 VOCs showed significant antifungal activities against six plant pathogenic fungi, namely, *Guignardia camelliae*, *Fusicoccus aesculin*, *Fusarium oxysporum*, *Alternaria tenuissima*, *Sphaeropsis sapinea*, and *Verticillium dahlia*. The fungi not exposed to *R. victoriana* JZ-GX1 were covered with plates, while the hyphae of all test fungi except *V. dahlia* in the presence of VOCs were limited to one side of the partition plate, and the pigments of *F. oxysporum* and *V. dahlia* were inhibited.

### Growth-Promoting Effect of *Rahnella victoriana* JZ-GX1 on Different Forest Trees in Field Trials

Eight months after inoculation, the microbial agent had a marked growth-promoting effect on the height and ground diameter of I-72 poplar compared with that of the CK, with growth rates of 9.7 and 16.1%, respectively. The growth rates of plant height and ground diameter of *P. elliottii* inoculated with strain JZ-GX1 were 23.6 and 14.6% greater than those of uninoculated trees, respectively. The height of pear trees with root application of strain JZ-GX1 was 14% higher than that of the control trees, but the treatment had no effect on ground diameter. For *M. spectabilis*, the plant height after inoculation increased to varying degrees compared with the ground diameter, but the difference was not significant (Table 4). These results of this field experiment support the discovery of multiple PGP mechanisms in the genome of *R. victoriana* JZ-GX1.

**TABLE 4:** Growth-promoting effect of *Rahnella victoriana* JZ-GX1 on different plants.

		Plant height (m)	Ground diameter (mm)
*P. euramericana* (Dode) Guinier cv. “san Martino” (I-72 poplar)	CK	4.55 ± 0.28a	39.90 ± 4.69a
	JZ-GX1	4.99 ± 0.20b	46.32 ± 3.54b
*P. elliottii*	CK	49.98 ± 13.49a	13.52 ± 3.79a
	JZ-GX1	61.79 ± 13.42b	15.49 ± 3.12b
*P. serotina*	CK	147.85 ± 12.99a	17.82 ± 1.70a
	JZ-GX1	168.53 ± 11.05b	17.81 ± 2.98a
*M. spectabilis*	CK	212.36 ± 7.86a	25.55 ± 3.07a
	JZ-GX1	215.20 ± 10.92a	26.10 ± 3.39a

*Means ± standard deviations (n = 90). Different letters indicated a significant difference between control and inoculation treatment based on student’s t-test (p < 0.05).*

## Discussion

Plant growth-promoting rhizobacteria have been demonstrated to produce the auxin IAA to enhance plant growth by controlling many physiological processes, such as cell division, vascular tissue differentiation and root elongation (Liu et al., 2019; Luziatelli et al., 2020). However, the biosynthetic pathways of IAA in bacteria are different. Five tryptophan-dependent IAA synthesis pathways have been identified in bacteria, namely, the indole-3-acetonitrile (IAN), tryptamine (TAM), indole-3-acetamide (IAM), indole-3-pyruvate (TPyA), and tryptophan side-chain oxidase (TSO) pathways, and a tryptophan-independent pathway (Zhang et al., 2019b). It has been reported that *R. aquatilis* ZF7 can synthesize IAA through the IPyA pathway (Yuan et al., 2020), and in this study, *R. victoriana* JZ-GX1 was predicted to contain two IAA production pathways (TPyA and IAM). PGPR usually interacts with plant root exudates and synthesizes IAA with the corresponding tryptophan as the precursor (Bhattacharjee et al., 2012; Liu et al., 2016). The JZ-GX1 strain has more than one IAA synthesis pathway, which indicates that it has a stronger ability to secrete IAA than the same microorganism.

Phosphorus (P) is one of the most limiting nutrients required for the growth and development of plants (Zeng et al., 2017; Li et al., 2018). Phytic acid mineralizing rhizobacteria (PMR) play an important role in promoting the dissolution of insoluble organic phosphorus in soil (Kour et al., 2021). *R. victoriana* JZ-GX1 was initially isolated with high phytate-degrading activity as an index, which can promote an increase in the total P content in maize (Li et al., 2013, 2021). However, the mechanism of organophosphorus dissolution has not been clearly revealed. The preliminary determination of the enzymatic properties of *R. victoriana* JZ-GX1 showed that it has a strong ability to hydrolyze phytate under acidic conditions (Li and Wu, 2014). In this study, we found that *R. victoriana* JZ-GX1 harbors two phytase-encoding genes (acid glucose-1-phosphatase and 4-phytase) but lacks appA-like phytase genes. The results of this study are consistent with previously reported enzymatic properties. Until now, there has been very little information available regarding the regulation of phytate-degrading gene expression in bacteria. The presence of phytase genes in this genome could open up opportunities for future molecular cloning and application studies of phytase from *R. victoriana* JZ-GX1.

In the rhizosphere, long-distance interactions through VOCs are an important method of signal transmission between bacteria and plants (Fincheira and Quiroz, 2018; Kong et al., 2021; Li et al., 2021). To date, many bacteria have been shown to stimulate plant growth by releasing volatiles. For example, *B. subtilis* SYST2-derived VOCs can promote tomato growth both *in vitro* and in pot experiments by triggering growth hormone activity (Tahir et al., 2017). Similar findings were obtained for *Arabidopsis* treated with volatiles from *B. megaterium* B55, and the combined effects of blended compounds increased leaf number and leaf surface area up to 2- and 4-fold, respectively, compared with those of the control (Meldau et al., 2012). *B. amyloliquefaciens* GB03 generates 3-hydroxy-2-butanone (acetoin) and 2,3-butanediol as its primary volatiles, which were shown to promote plant growth in trials on *Arabidopsis* (Ryu et al., 2003). The role of these two compounds in plant growth promotion was also confirmed by the addition of the pure acetoin and a mutant bacterium lacking a 2,3-butanediol biosynthesis gene (Farag et al., 2006). In our research, we also detected acetoin in the JZ-GX1 strain by gene prediction and physiological detection, and confirmed that JZ-GX1-derived VOCs could promote an increase in fresh weight and root development without contact with *A. thaliana*. Although the specific mechanism needs to be further explored, this is the first report on the biological activity in plant growth promotion of the VOCs produced by *Rahnella* sp.

When plants are faced with abiotic stress, several important metabolites, including osmotic regulators and active molecules, accumulate in large quantities (Zhou et al., 2017). Spermidine (Spd) is an important plant growth regulator and has also been identified as a protector against various abiotic stressors, such as high salinity, drought, low temperature, and iron deficiency (Zhou et al., 2016a; Li G. et al., 2020; Qian et al., 2021). Reportedly, *B. subtilis* STU6 induces and uses plant arginine to produce more Spd, which promotes bacterial colonization and absorption by the host, thus enhancing the iron acquisition system and cell wall iron remobilization in a NO-dependent manner (Zhou et al., 2019). Three genes of spermidine synthase (GE00638, GE00674, and GE00675) were identified in strain JZ-GX1. Furthermore, GABA plays a vital role in plant responses to multiple stressors (Al-Quraan et al., 2013). Guo et al. (2020) found that GABA can improve the tolerance of cucumber to iron deficiency in an auxin-dependent manner. In a previous study, we demonstrated the potential roles of *R. victoriana* JZ-GX1 in alleviating iron deficiency-induced chlorosis in *C. camphora* (Kong et al., 2020b). Here, strain JZ-GX1 was found to possess the genes encoding glutamate decarboxylase, which is involved in GABA production and degradation (GE04524, GE04546, and GE04695). Combined with the previous results, we further clarified the potential mechanism by which *R. victoriana* JZ-GX1 promotes iron uptake in plants. However, the contents of these two substances in plants should be determined, and the genes for the synthesis of these two substances by *R. victoriana* JZ-GX1 should be knocked out to prove this conjecture.

At present, the genus *Rahnella* consists of six closely related species, including *R. aquatilis*, *R. variigena*, *R. victoriana*, *R. bruchi*, *R. woolbedingensis*, and *R. inusitata* (Yuan et al., 2020). Among them, *R. aquatilis* has been widely reported to promote plant growth (Peng et al., 2019; Zhang et al., 2019a; Sun et al., 2020). There are few reports regarding the other species, and so far the only reports of *R. victoriana* was considered associated with decline symptoms on oak and hornbeam (Yousef et al., 2019). In this study, *R. victoriana* JZ-GX1 was proven to be a PGPR strain with a variety of beneficial features, including IAA production, phosphate solubilization, nitrogen fixation, siderophores, acetoin, ACC deaminase, GABA production, spermidine and VOCs biosynthesis. In addition, the ability of strain JZ-GX1 to promote forest growth has also been well-verified in complex field environments. Overall, the data obtained in this study provide more clues for the potential application of *R. victoriana* JZ-GX1 in the development of eco-friendly biofertilizers, which can promote soil fertility and crop yield.

## Data Availability Statement

The datasets presented in this study can be found in online repositories. The names of the repository/repositories and accession number(s) can be found at: https://www.ncbi.nlm.nih.gov/, PRJNA720502.

## Author Contributions

W-LK completed the data analysis and the first draft of the manuscript. W-LK, W-YW, and S-HZ were the finishers of the experimental research. W-YW participated in the experimental result analysis. X-QW directed experimental design, data analysis, and manuscript writing and revision. All authors read and agreed on the final version of manuscript.

## Conflict of Interest

The authors declare that the research was conducted in the absence of any commercial or financial relationships that could be construed as a potential conflict of interest.

## Publisher’s Note

All claims expressed in this article are solely those of the authors and do not necessarily represent those of their affiliated organizations, or those of the publisher, the editors and the reviewers. Any product that may be evaluated in this article, or claim that may be made by its manufacturer, is not guaranteed or endorsed by the publisher.

## Footnotes

1
http://kbase.us

2
https://www.ezbiocloud.net/tools/ani
