# Supplementary material for: Genome Sequencing of Rahnella victoriana JZ-GX1 Provides New Insights Into Molecular and Genetic Mechanisms of Plant Growth Promotion
Source: Front Microbiol. 2022 Mar 30;13:828990. doi: 10.3389/fmicb.2022.828990 (PMC9020876; doi:10.3389/fmicb.2022.828990)
Supplement: Supplementary file 1 [file Table_1.DOCX]

Table S1 Oligonucleotide primers used in this study.

| Primer | Sequence | Target | Size  (bp) |
| --- | --- | --- | --- |
| acdS-F | ATGATGAAACAAGCAGATGATTTTG | ACC deaminase | 1002 |
| acdS-R | TCAATGCTCCAGCAAGGTTTG |  |  |
| gadB-F | ATGTCCGAGTTAAACCCGATTC | GABA production | 1473 |
| gadB-R | TTACTGGTTCAGCAGTTGCTGC |  |  |
| gabD-F | ATGACCACTGCCAGCACACA |  | 1392 |
| gabD-R | CTACTGACGGTTGCTCCACACG |  |  |
| gabT-F | ATGAGTCACAGTGAACTGGAAAAACG |  | 1269 |
| gabT-R | TCAGTCAGCCAGTACGTCGGA |  |  |
| speA-F | ATGTCTGATGACATCCAGTCCCA | Spermidine biosynthesis | 1983 |
| speA-R | TTATTCGTCTTCTTCCAGATACGTATAA |  |  |
| speD-F | TTGCAAAAGCTGAAACTGCATG |  | 795 |
| speD-R | TCAGACTGACGGCAGATTGCG |  |  |
| nirB-F | ATGAGCAAAGTCAGACTCGCG | Nitrite reductase | 2550 |
| nirB-F | TCATACTTCCTCCCCGTCAGTG |  |  |
| PstA-F | ATGATTGAAAACCGCAGAGGG | Phosphate transport protein | 846 |
| PstA-R | TTACTTCTCACTGTCTACCAGACCGC |  |  |
| PstC-F | ATGGCTGAATACAAGCCGGC |  | 957 |
| PstC-R | TTAACGGCCCTCTTTCTTAGCC |  |  |
| G1Pase-F | GTGAAAAAAGCATCCCGTTTTC | Glucose-1-phosphatase | 1302 |
| G1Pase-R | TCAGCCTTGCAGATCCTTTTG |  |  |

Table S2 Genomic features, gene prediction, and annotation summary of the JZ-GX1 genome.

| Features | Value |
| --- | --- |
| Genome size (bp) | 5,472,828 |
| GC content (%) | 53.53 |
| Plasmid | 2 |
| Coding Region (bp) | 4,788,741 |
| Genes assigned to COG | 88.85% |
| Genes assigned to KEGG | 68.77% |
| Genes assigned to GO | 80.68% |
| rRNA | 22 |
| tRNA | 77 |
| other ncRNA | 83 |
| Pesudogene number | 0 |
| CRISPR | 12 |
| Geneisland | 12 |
| prophage | 2 |
